# Adenovirus E1A/E1B Transformed Amniotic Fluid Cells Support Human Cytomegalovirus Replication

**DOI:** 10.3390/v8020037

**Published:** 2016-02-02

**Authors:** Natascha Krömmelbein, Lüder Wiebusch, Gudrun Schiedner, Nicole Büscher, Caroline Sauer, Luise Florin, Elisabeth Sehn, Uwe Wolfrum, Bodo Plachter

**Affiliations:** 1 Institute for Virology, University Medical Center of the Johannes Gutenberg-University Mainz, D-55131 Mainz, Germany; kroemme@uni-mainz.de (N.K.); bueschni@uni-mainz.de (N.B.); sauer.caroline@gmx.de (C.S.); 2 Department of Pediatric Molecular Biology, Charité University Medical Centre Berlin, D-10117 Berlin, Germany; lueder.wiebusch@charite.de; 3 CAP-CMV GmbH, D-51105 Cologne, Germany; schiedner@t-online.de; 4 Institute for Medical Microbiology and Hygiene, University Medical Center of the Johannes Gutenberg-University Mainz, D-55131 Mainz, Germany; lflorin@uni-mainz.de; 5 Institute for Zoology, Johannes Gutenberg-University Mainz, D-55099 Mainz, Germany; sehn@uni-mainz.de (E.S.); wolfrum@uni-mainz.de (U.W.); 6 Research Center for Immunotherapy, University Medical Center of the Johannes Gutenberg-University Mainz, D-55131 Mainz, Germany

**Keywords:** cytomegalovirus, amniotic fluid cells, adenovirus E1A/E1B, CAP, Cevec’s aminocyte production cell line

## Abstract

The human cytomegalovirus (HCMV) replicates to high titers in primary human fibroblast cell cultures. A variety of primary human cells and some tumor-derived cell lines do also support permissive HCMV replication, yet at low levels. Cell lines established by transfection of the transforming functions of adenoviruses have been notoriously resistant to HCMV replication and progeny production. Here, we provide first-time evidence that a permanent cell line immortalized by adenovirus type 5 E1A and E1B (CAP) is supporting the full HCMV replication cycle and is releasing infectious progeny. The CAP cell line had previously been established from amniotic fluid cells which were likely derived from membranes of the developing fetus. These cells can be grown under serum-free conditions. HCMV efficiently penetrated CAP cells, expressed its immediate-early proteins and dispersed restrictive PML-bodies. Viral DNA replication was initiated and viral progeny became detectable by electron microscopy in CAP cells. Furthermore, infectious virus was released from CAP cells, yet to lower levels compared to fibroblasts. Subviral dense bodies were also secreted from CAP cells. The results show that E1A/E1B expression in transformed cells is not generally repressive to HCMV replication and that CAP cells may be a good substrate for dense body based vaccine production.

## 1. Introduction

Human cytomegalovirus (HCMV) remains associated with considerable disease burden, despite the availability of antiviral medication [1]. HCMV infection during pregnancy may result in intrauterine transmission with congenital infection rates ranging between 0.05% and 3.6% in newborns, depending on the characteristics of the maternal population [2,3,4,5]. Roughly 10% of congenitally infected infants will develop long-term sequelae [6,7]. Intrauterine growth restriction by placental HCMV infection may, in addition, occur, regardless of virus transmission to the fetus [8,9]. Further to that, HCMV infection still is a major complication in solid organ transplant or hematopoietic stem cell recipients [10,11,12]. All of that has fostered attempts to develop therapeutic or prophylactic strategies to cope with the consequences of HCMV infection [13,14,15].

Recent work focused on the use of inactivated whole virus or subviral particles as a basis for HCMV vaccine development [14,16,17,18,19]. Of particular interest is the use of HCMV dense bodies (DBs) for that purpose [14,19,20]. These particles are synthesized following HCMV infection of fibroblast cultures. Besides the limited availability of human fibroblasts that are licensed for vaccine production, these cells require the addition of fetal calf serum for growth. A number of other cell types are also permissive to HCMV infection, yet to limited levels [21,22,23,24]. In addition, all of these cells grow in an anchor-dependent manner, rendering them challenging for cost-effective and safe vaccine production processes. One strategy to gain high-level, permanently growing cell lines for vaccine production is immortalization with adenoviral early gene functions E1A and E1B. Human cells, however, are not easily transformed by E1A and E1B. Transformation of human embryonal kidney cells with E1A and E1B of adenovirus type 5 (Ad5) resulted in one single clone [25]. These HEK293 cells have been widely used for research purposes as well as for commercial applications (reviewed in [26]), yet they do not support the full HCMV replication cycle [27,28]. Transformation of primary human fetal retinoblastoma cells with E1A/E1B of Ad5 led to the establishment of various cell lines (HER), one of which (PER.C6) has been used for commercial purposes [26,29,30]. However, propagation of HCMV on PER.C6 cells has not been reported.

Recently, a cell line was established from cells of fetal amniotic fluid by transformation with the early gene functions E1A and E1B of Ad5 [31]. These cells (CAP; Cevec’s aminocyte production cell line; CEVEC Pharmaceuticals, Cologne, Germany) grow both anchor-dependent and in suspension and do not require animal serum for culture. CAP cells have been successfully used to grow viruses like influenza, Respiratory Syncytial Virus, or poliovirus [32,33,34], as well as to express heterologous proteins [26,35]. Pilot experiments exposing CAP cells to HCMV in our laboratory provided surprising results, indicating a high level of initial viral infection and gene expression, leading to marked cytopathic alterations. This was opposed to what was known from other human cells transformed with E1A/E1B. This property rendered CAP cells of interest as a substrate for vaccine production. We thus decided to investigate the susceptibility of CAP cells towards HCMV infection and DB-production. The experiments showed that CAP cells supported the whole replication cycle of HCMV and DB-release, rendering them exceptional to other E1A/E1B transformed cell lines.

## 2. Materials and Methods

### 2.1. Cells

CAP cells were cultured in suspension (CAP_sus._) under serum free conditions in CAP-T Express medium (Biochrom, Berlin, Germany), supplemented with 6 mM l-alanyl-l-glutamine (Biochrom) and 50 mg/L gentamycin (Life Technologies, Darmstadt, Germany), or were grown on support (CAP_adh._) in OptiPro™ SFM (Life Technologies), supplemented with 10% (*v/v*) fetal calf serum (FCS; Biochrom), 4 mM l-alanyl-l-glutamine (Biochrom) and 50 mg/L gentamycin (Life Technologies). CAP_sus._ were grown in Erlenmeyer flasks with vent cap (Corning Inc., Kaiserslautern, Germany). CAP_adh._ were grown in cell culture flasks (Greiner Bio-One International, Frickenhausen, Germany) at 37 °C. Human embryonic kidney cells HEK293 (CRL-1573; ATCC) were cultivated in DMEM (Sigma Aldrich, Munich, Germany), supplemented with 10% (*v/v*) FCS (Biochrom), 2 mM l-glutamine (Sigma Aldrich), 1× MEM non-essential amino acids solution (Life Technologies), 1 mM sodium pyruvate (Life Technologies), and 50 mg/L gentamycin (Life Technologies). The embryonic lung fibroblast cell line MRC5 (#19; Frauenhofer Institute, Leipzig, Germany) and human foreskin fibroblasts (HFF, line Psf5) were cultivated in MEM (Life Technologies), supplemented with 10% (*v/v*) FCS (Biochrom), 2 mM l-glutamine (Sigma Aldrich), 0.5 ng/mL bFGF (Life Technologies), and 50 mg/L gentamycin (Life Technologies). HEK293, MRC5, and HFF were cultivated in cell culture flasks (Greiner Bio-One International) at 37 °C.

### 2.2. Viruses

For infection, HB5 [36], Towne-BAC [37,38], TB40/E derived HCMV strains TB40/E-deltaUL16EGFP [39]; TB40/E-BAC7 [40], TB40/E-BAC4deltaUL5-9luc [41] or a UL130 repaired variant of the Towne laboratory strain (TowneUL130rep) were used. The strains HB5 and Towne-BAC represent the laboratory strains AD169 and Towne which were cloned as bacterial artificial chromosomes (BAC) [36,37,38]. Towne-BAC expresses the green fluorescent protein (GFP) under simian vacuolating virus 40 (SV40) early promotor control. TB40/E-BAC7 is a BAC-clone derived strain, based on the recent isolate TB40/E [40]. TB40/EdeltaUL16EGFP is a viral strain that expresses GFP under the control of the HCMV UL16 early promotor. Insertion led to the truncation of the UL16 open reading frame. Cloning of the respective BAC was performed in Christian Sinzgers laboratory in analogy to AD169deltaUL16GFP, as described by Digel *et al.* [39]. TB40/E-BAC4deltaUL5-9luc is a TB40/E-derived viral strain that lacks the genomic region encoding UL5-9. The region was replaced by a gene encoding the firefly luciferase under SV40 early promotor control [41]. For most of the experiments, Towne-BAC and TowneUL130rep were used. These strains are genetically identical except for a mutation in UL130 in Towne-BAC which is repaired in TowneUL130rep to allow the expression of pUL130 and consequently the formation of the pentameric complex gH-gL-gpUL128-131A. Both of these strains express GFP. Virus stocks were generated on HFF. The infectivity contained in these stocks was determined on HFF in 96-Well plates by serial dilution of the supernatants and staining for IE1-positive cells after a 48 h-infection. Staining was done with the IE1-specific monoclonal antibody (mAb) p63-27 [42] in eight technical replicates. The infectivity contained in these stocks was calculated as the number of IE1-positive cell-inducing units per volume (mL) of stock solution (culture supernatant; see Section 2.8 for details). Based on that value, an m.o.i. was defined, *i.e.*, the number of IE1-positive cell inducing units that were applied per cell (CAP_sus._, CAP_adh._, HEK293, MRC5 and HFF) in the respective experiment.

### 2.3. Analysis of Viral Protein Expression in Suspension CAP Cells

5 × 10^5^/mL CAP_sus._ were cultured in Erlenmeyer flasks with vent cap at 37 °C, 260 rpm overnight. Following that, cells were infected with TowneUL130rep at an m.o.i. of 0.5. For this, cells were centrifuged (150× *g*, 5 min, RT) and were then resuspended in medium, containing TowneUL130rep in a concentration to result in the desired m.o.i. After that, cells were delivered to the flask and were shaken at 50 rpm for 2 h under humid conditions. Then, culture medium was added and the flasks were shaken at 200 rpm until further use. One day, two days and three days after infection, cytospin slides of infected and mock-infected cells were prepared. For this, 1 × 10^4^–1 × 10^5^ cells were collected, centrifuged, resuspended in 100 µL 1× phosphate buffered saline (PBS) and transferred into Schandon Cytoslides (Thermo Fisher Scientific, Darmstadt, Germany). After centrifugation in a Cytospin 4 cytocentrifuge for 5 min, the slides were air dried for 30 min. Afterwards, cells were fixed in a 1:1 acetone:methanol mixture and were again air dried. To investigate expression of particular viral proteins, slides were stained with specific antibodies, using the alkaline-phosphatase-anti-alkaline-phosphatase (APAAP) technology (Dako, Hamburg, Germany). For staining of pp65, the Clonab CMV antibody (Biotest, Dreieich, Germany) was used in a dilution of 1:10 in 1× tris-buffered saline (TBS), containing 1% bovine serum albumin (BSA). For staining of IE1, undiluted hybridoma supernatant of the monoclonal antibody p63-27 (donation of William Britt) was used. Twenty-five microliters of each dilution were applied to the cytospin spots. Incubation lasted 30 min at room temperature in a humid chamber. After incubation, slides were washed two times in 1× TBS for 5 min each. In a second step, bridging antibody anti-mouse-Ig (M5899; Sigma-Aldrich) was applied at a dilution of 1:25 in 1× TBS with 1% BSA. Twenty-five microliters of this solution was applied to each spot. Incubation was for 30 min at room temperature in a humid chamber. After incubation, slides were washed again two times in 1× TBS for 5 min each. In a next step, the antibody from the APAAP system (A7827; Sigma-Aldrich) was diluted 1:50 in 1× TBS with 1% BSA. Twenty-five microliters of that solution was applied to each spot and incubated for 30 min at room temperature. After incubation, slides were washed two times in 1× TBS for 5 min each. For staining, the Fuchsin + substrate-chromogen system (Dako) was added as specified by the manufacturer. After staining, the slides were rinsed three times in 1× TBS and once with distilled water. Slides were then incubated in filtrated haematoxilin (Merck, Darmstadt, Germany) for 1 min and were then incubated in tap water for 5 min. Spots were covered with 10–12 mm cover slips using mounting medium.

### 2.4. Indirect Immunofluorescence (IF)

For all indirect immunofluorescence analyses, 5 × 10^5^ HFF or 1 × 10^6^ CAP_adh._ were seeded in 10 cm culture dishes (78 cm^2^). Coverslips were inserted into these dishes. Following overnight incubation, cells were infected with either HB5 at an m.o.i. of 1, Towne-BAC at an m.o.i. of 1, or TB40/E-BAC7 at an m.o.i. of 2. For investigation of viral nuclear replication compartments, CAP_adh._ were incubated two days before infection with TB40/E-BAC7 at an m.o.i. of 0.5. A mock-infected control was carried along for each cell type. Depending on the experimental setup, cover slips were collected either 3 h, 5 h, 1 d, 2 d, or 5 d after infection, washed with 1× PBS and fixed in acetone p.a. for 20 min at room temperature. Subsequently, the cell membrane was permeabilized by several rounds of rinsing in 1× PBS/0.1% TritonX (Roth, Karlsruhe, Germany). In the case of infection with the laboratory strains, cells were incubated with murine mAb p63-27 [42] for 1 h, 37 °C. For investigation of viral nuclear replication compartments, cells were incubated with a murine mAb directed against pUL44 (BS510, Biotest). For the ND10 domain experiment, cells were incubated with either murine mAb p63-27 [42], rabbit polyclonal anti-PML serum (Santa Cruz, Heidelberg, Germany) or both for 1 h, 37 °C. Cells were then again washed in 1× PBS/0.1% TritonX (3 times). Depending on the experimental setup, cells were either incubated with a FITC- or an Alexa Fluor 488-labelled secondary antibody, directed against mouse immunoglobulin and/or an Alexa Fluor 546-labelled secondary antibody directed against rabbit immunoglobulin for 1 h, 37 °C (Santa Cruz). For staining of the nuclei, DAPI, diluted in 1× PBS, was added to the cover slips. Cells were then kept at RT in the dark for 10 min. After that, cover slips were rinsed several times in 1× PBS/0.1% TritonX and once with H_2_O_dest._. After that, coverslips were transferred to glass slides and fixed with Mowiol. Glass slides were kept in the dark at room temperature to allow drying until inspection. Images were acquired using the Zeiss Axiovert 200 M microscope equipped with a Plan-Apochromat 100 objective (1.4 NA) and the Axiovision deconvolution software 4.7 (Zeiss, Jena, Germany).

### 2.5. Flow Cytometry of Viral Protein Expression

Protein expression of infected CAP_adh._ was measured by flow cytometry (FACS) according to Wiebusch and Hagemeier [43]. Briefly, CAP_adh._ were seeded in cell culture flasks and were incubated over night at 37 °C. The following day, cells were infected with TB40/E-BAC4delUL5-9luc at an m.o.i. of 2. The inoculum was removed 1.5 h after the addition of virus. Cells were then washed with 0.14 M NaCl and were then exposed to 0.1 M glycine (pH 3.0) to inactivate residual virus. Afterwards cells were again incubated at 37 °C. At 3 d after infection, cells were collected and fixed with ethanol. Cells were then incubated over night with specific mAbs directed against either IE1 (p63-27; [42]) or pp28 (Santa Cruz). The next day, cells were washed with 1× PBS/1% BSA and were then incubated with a secondary antibody, conjugated to fluorochrome AF647 against mouse IgG (Life Technologies). Cells were then analyzed via flow cytometry using the Cytomics FC 500 apparatus from Beckman Coulter.

### 2.6. Quantitative PCR Analysis of HCMV Genome Replication

Normalization of input virus was performed by determining the genome copy number in 6 h infected cells. For this, 0.5 × 10^5^ CAP_adh._ and 0.5 × 10^5^ HFF were seeded in 10 cm dishes (20 dishes total, 5 each per cell type and per viral strain). After overnight incubation, cells were infected in 3 mL culture medium, using different virus dilutions (5 µL, 10 µL, 50 µL, 100 µL, 500 µL) of culture supernatant from HB5- and Towne-BAC-infected HFF. After an adsorption period of 1.5 h, dishes were replenished with additional 7 mL of culture medium. At 6 h after infection, the supernatant was discarded. Cells were washed two times using 1× PBS. Following that, trypsin was added to HFF, spread over the dish and left for 5 min at 37 °C. For CAP_adh._, trypsin was applied, spread and discarded following an incubation of 5 min at 37 °C. After that, FCS containing medium was added to stop the reaction. Following that, cells were detached from the support and were centrifuged for 5 min. The cell pellet was resuspended in 1× PBS. Cells were counted and adjusted to 1 × 10^6^/mL. DNA was isolated using the “High Pure Viral Nucleic Acid Kit” (Roche) according to the manufacturer’s instructions. Viral DNA concentration was determined in each sample using the ABI 7500 Fast Real-Time PCR Detection System. Amplification was done as follows: 2 min/50 °C, 5 min/95 °C, 45 cycles of 94 °C/15 s, 60 °C/1 min. The results were calculated in genome copies/cell.

### 2.7. Replication Kinetics and Release of Viral Genomes into the Cell Culture Supernatant of Infected CAP Cells

In 9 × 75cm^2^ culture dishes, 0.5 × 10^6^ HFF (positive control), 0.5 × 10^6^ HEK293 (negative control) or 1 × 10^6^ CAP_adh._ were seeded. Ten milliliters of the appropriate culture medium was added. Incubation was overnight at 37 °C in humid conditions. Cells were infected with TowneUL130rep the following day. Infection was with an m.o.i. of 2.5. At 1.5 h after the addition of virus, the inoculum was removed. One milliliter of the inoculum of each dish served as sample 0 d post infection for viral genome release. The cells of these dishes, sample of 0 d post infection in HCMV replication analysis, were washed with 1× PBS, detached from the dish by trypsin, centrifuged, counted and adjusted to 1 × 10^6^/mL with 1× PBS. Afterwards, the residual cell culture dishes were washed with 0.14 M NaCl, exposed to 0.1 M glycine (pH 3.0) for 1 min to inactivate residual virus, and washed with maintenance medium. Cells were then either incubated with or without the addition of 0.25 mg/mL PAA at 37 °C. Cells and viral supernatants were collected at 2 d, 4 d and 6 d after infection. Samples were stored at −20 °C until further use. The DNA contained in 100 µL of each cell suspension sample or 200 µL of each viral supernatant was extracted using the “High Pure Viral Nucleic Acid Kit” (Roche) according to the manufacturer’s instructions. Quantification of viral DNA was performed using the ABI 7500 Fast Real-Time PCR Detection System. Results were either calculated in genome copies per cell or genome copies per mL.

### 2.8. Release of Infectious HCMV into the Culture Supernatant of Infected CAP Cells, Measured by IE1-Staining

HFF and CAP_adh._ were infected with TB40/E-deltaUL16EGFP at an m.o.i. of 10 (CAP_adh._) or 1 (HFF), respectively. Then, 6 d, 8 d, 10 d and 13 d after infection, supernatants were collected, cleared by centrifugation and stored in aliquots at −80 °C. Determination of infectivity was performed by staining with a mAb directed against IE1 (p63-27; [42]). For this, 5 × 10^3^ HFF were seeded per well on a 96-well plate. Cells were infected with the supernatants the following day. For this, samples were thawed and diluted by 10^1^–10^4^. One hundred microliters of each were applied in 8 biological replicates to the cells in the 96-well plates. Forty-eight hours after infection, cells were rinsed in 1× PBS and were subsequently fixed with 96% ethanol for 20 min at room temperature. After that, cells were again rinsed in 1× PBS and were incubated for 1 h at 37 °C with 50 µL hybridoma supernatant of the IE1-specific monoclonal antibody (p63-27; [42]) under humid conditions. Binding of the IE1-specific mAb was measured by using a horseradish peroxidase labelled secondary antibody against murine immunoglobulin (Dako). Antibody complexes were visualized by incubation of the cells with a 3-amino-9-ethylcarbazole (AEC) solution. Afterwards, cells were again rinsed in 1× PBS to stop the enzymatic reaction and were then covered with 1× PBS and stored at 4 °C until microscopic inspection. IE1-positive nuclei in the 8 biological replicates were counted. The mean of that was taken as relative measure of infectivity.

### 2.9. Electron Microscopy

Transmission electron microscopy was performed as described previously [44]. Briefly, 1 × 10^6^ CAP_adh._ and 0.5 × 10^6^ HFF (control) were seeded in 10 cm dishes. After overnight incubation, cells were infected in 3 mL culture medium at an m.o.i. of 5, 10 (CAP_adh._), or 1 (HFF), using TowneUL130rep or TB40/E-BAC4deltaUL5-9luc. Then, 1.5 h after the addition of virus, the inoculum was removed. Cells were washed with 0.14 M NaCl, exposed to 0.1 M glycine (pH 3.0) for 1 min to inactivate residual virus, and washed with maintenance medium. Afterwards cells were again incubated at 37 °C. At 3 d, 6 d or 8 d after infection, culture medium was removed and cells were washed once with 1× PBS. Five milliliters of trypsin were added and left on the cells at 37 °C for 5 min. Addition of medium with FCS was used to stop the reaction. Cells were then centrifuged and 1× PBS was added afterwards (to wash the cells again). Cells where subsequently fixed with 2.5% glutaraldehyde/0.1 M cacodylate buffer/0.1 M sucrose. After an incubation period of 2 h, cells were washed three times with 0.1 M cacodylate buffer/0.1 M sucrose. Another fixation step was performed with 2% OsO_4_ in 0.1 M cacodylate buffer/sucrose for 1 h. Cells were then resuspended in agar. Residual water was extracted from cells by washing with increasing concentrations of ethanol (30%, 50%, 70%, 80%, 96%, 100%). Afterwards, cells were washed two times with propylene oxide before they were embedded in a 1:1 mixture of propylene oxide and araldite overnight. As a last step, cells were polymerized with araldite for 48 h, 65 °C. Ultrathin slices were made and analyzed by TEM [44,45].

### 2.10. Analysis of Dense Bodies (DBs) Production from Infected CAP Cells

For DB-purification, 1.8 × 10^6^ CAP_adh._ were seeded in 20 × 175 cm^2^ tissue culture flasks. Twenty milliliters of the appropriate culture medium was added to each flask. Afterwards, 1.8 × 10^6^ HFF were seeded in 10 × 175 cm^2^ tissue culture flasks and were taken along as control. In the case of CAP_adh._, incubation lasted 48 h at 37 °C under humid conditions. HFF were incubated only overnight under humid conditions. One day (HFF) or two days after seeding (CAP_adh._), cells were infected with TowneUL130rep at an m.o.i. of 1 (HFF), 2.5 or 5 (CAP_adh._). One day after infection, a complete medium exchange was performed. After 7 days, the culture supernatants were collected. Supernatants of ten flasks each were combined and centrifuged (1300× *g*, 10 min, RT). After that, the supernatant was collected and centrifuged at 100,000× *g* (70 min, 10 °C) in a SW32Ti rotor in a Beckman Optima L-90K ultracentrifuge. Meanwhile, the gradients were prepared by mixing 4 mL 35% Na-tartrate solution with 5 mL 15% Na-tartrate/30% glycerin-solution in 0.04 M sodium-phosphate buffer pH 7.4, using a gradient mixer and Beckman Ultra-clear^TM^ centrifuge tubes (14 × 89 mm). Following centrifugation, the pellets were resuspended in 1000 µL 1× PBS. The suspension was applied on top of one gradient. Centrifugation was performed at 91,000× *g* (60 min, 10 °C) in a SW41 rotor. After centrifugation, the bands, corresponding to Non-Infectious Enveloped Particles (NIEPs), virions and DBs were visualized by light scattering and collected from the gradient, using a syringe and an 80 G × 1.5”-gauge needle. Each sample was supplemented with 1× PBS to give a total volume of 10 mL. Centrifugation was then performed at 99,000× *g* (90 min, 10 °C) in a SW41 rotor. Following centrifugation, the pellets were resuspended in 50 µL (virions, DBs) or 100 µL (NIEPs) 1× PBS. Fifteen microliters were taken for the determination of the protein content, and the other samples were stored in aliquots at −80 °C until further use. The protein concentrations in the samples were evaluated by using the Pierce BCA protein assay kit (Thermo Scientific, order-No.: 23225) according to the manufacturer’s instructions. Then, a 10% SDS-polyacrylamide gel was used for the separation of the proteins in the samples. Two micrograms of each sample was used. Silver staining of the proteins was done using the Roti^®^-Black P-Silberfärbungskit für Proteine (Roth, order-No. L533.1) according to the manufacturer’s instructions.

## 3. Results

### 3.1. CAP Cells Support IE- and pp65-Gene Expression

**Figure 1:**
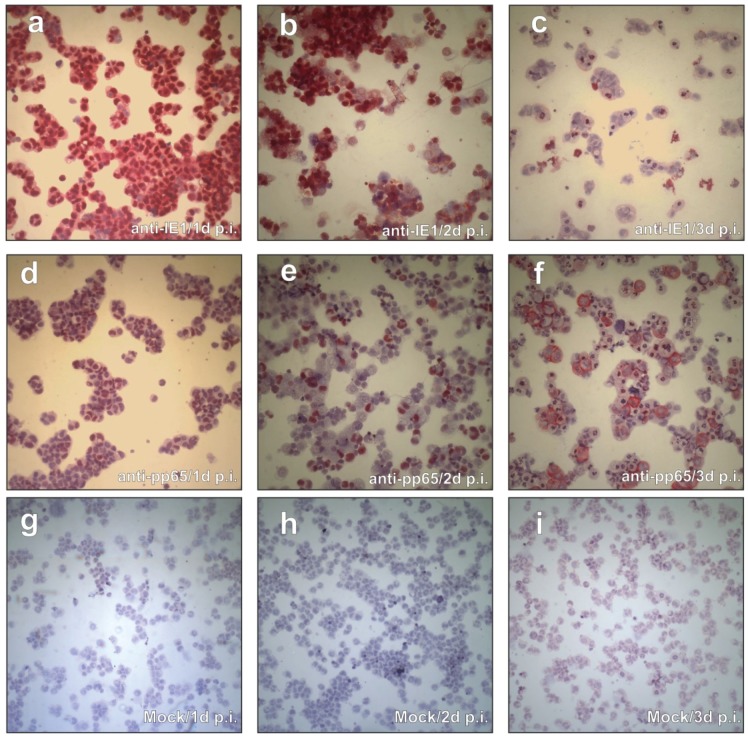
Viral protein expression in CAP_sus._ (**a**–**f**), cytospin preparations of HCMV infected CAP_sus._, stained with mAbs against IE1 (**a**–**c**) and pp65 (**d**–**f**). Cells were infected with TowneUL130rep at an m.o.i. of 0.5. (**g**–**i**), mock-infected cells. Cytospin preparation and staining was performed at the indicated times after infection. Red color depicts specific staining of the viral proteins.

In an initial attempt to test the susceptibility of CAP cells for HCMV infection, CAP_sus._ were exposed to TowneUL130rep. This virus expresses the viral envelope glycoprotein complex gH/gL/gpUL128-131A (pentameric complex) required for viral entry in cell types such as endothelial (EC) or epithelial cells [46,47]. At 1, 2, and 3 days after infection (d p.i.), cytospin samples were prepared and stained with mAbs against viral IE1 (pUL123; Figure 1a–c) and pp65 (ppUL83; Figure 1d–f). Close to 100% of the CAP_sus._ expressed IE1 at 1 d p.i. (Figure 1a). Since an m.o.i. of 0.5, HFF was used for this assay, this suggested that the efficiency of initial infection in CAP cells was higher compared to fibroblasts. Some of the cells were also faintly stained for pp65 at this time. This stain either originated from input particles or from *de-novo* synthesis of the tegument protein (Figure 1d). At 2 d p.i., still most of the cells were IE1-positive (Figure 1b). A fraction of the cells now displayed distinct pp65 expression in the nucleus (Figure 1e). At 3 d p.i., most of the intact cells did not show IE1 expression (Figure 1c). Yet, there appeared to be a number of irregularly shaped structures that showed IE1 staining. They most likely originated from lysed cells that still retained some of the IE1, possibly associated with chromatin [48]. Interestingly, the cells that stained for pp65 now showed a cytoplasmic localization of the tegument protein (Figure 1f), closely resembling the known pp65 localization in late-stage infected permissive fibroblasts.

Taken together, these data showed that CAP cells could be efficiently infected with HCMV and that they supported viral gene expression. We thus were interested in further studying the biology of HCMV infection in these cells. At this point, we switched to CAP_adh._ for our experiments, as these cultures were more comparable to adherent HFF, which served as controls. In addition, most of the assays that were employed were pre-established for HFF. 

**Figure 2:**
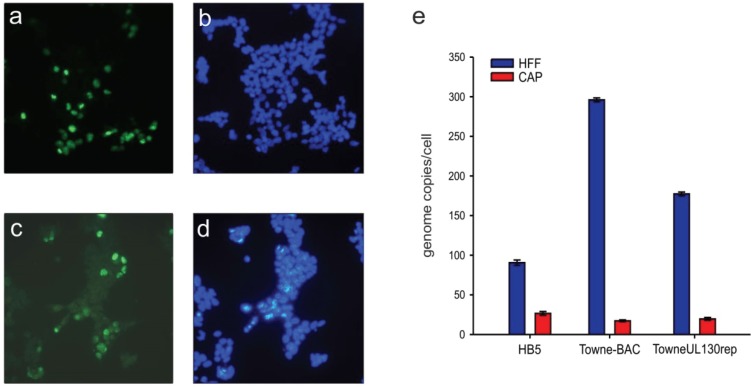
IF-analysis and viral genome uptake of CAP_adh._, infected with HCMV laboratory strains. CAP_adh._ were infected with strains Towne (Towne-BAC; **a**,**b**) or AD169 (HB5; **c**,**d**), respectively at an m.o.i. of 1. 2 d p.i., cells were stained for the expression of IE1 by IF (**a**,**c**) or were stained by DAPI (**b**,**d**); Genome uptake following infection with Ad169 (HB5), Towne (Towne-BAC) or TowneUL130rep (control) was measured 6 h p.i. by quantitative PCR analysis (**e**). The genome uptake of HFF, infected with the respective strains was taken along for control. Error bars represent means of three technical replicates.

Infection of CAP_adh._ with a TB40/E derivative of HCMV resulted in a distinct cytopathic effect [49]. To test if CAP_adh._ could also be infected with HCMV laboratory strains, infection was performed with BAC derivatives of the AD169 (HB5) or of the Towne (Towne-BAC) strain, respectively. These strains have been found to be severely restricted in their host cell range *in vitro* because of their failure to express the pentameric envelope glycoprotein complex composed of gH/gL and gpUL128-131A [23,46]. Following fixation with acetone, an indirect immunofluorescence analysis was performed using a mAb against the IE1 protein. Although Towne-BAC expresses GFP, this virus could be used in this case as acetone quenches the GFP signal. Surprisingly, a substantial number of CAP_adh._ stained positive for IE1, indicating that the infection could proceed independent of the pentameric glycoprotein complex (Figure 2). To evaluate the efficiency of infection, CAP_adh._ and, for control, HFF were infected with either HB5 or Towne-BAC and were analysed for the uptake of viral genomes. For control, cells were also infected with the pentamer-positive strain TowneUL130rep. For AD169, roughly 29% of the genomes that entered HFF were also found in CAP_adh._. For Towne, infection appeared to be less efficient, adding up to roughly 5.7%. The respective rate for Towne-rep was 11.1%. These results showed that, opposed to EC or epithelial cell cultures, CAP_adh._ infection by HCMV was at least partially independent of gH/gL/gpUL128-131A.

### 3.2. Cellular PML-Bodies Are Dissolved in HCMV-Infected CAP Cells

**Figure 3:**
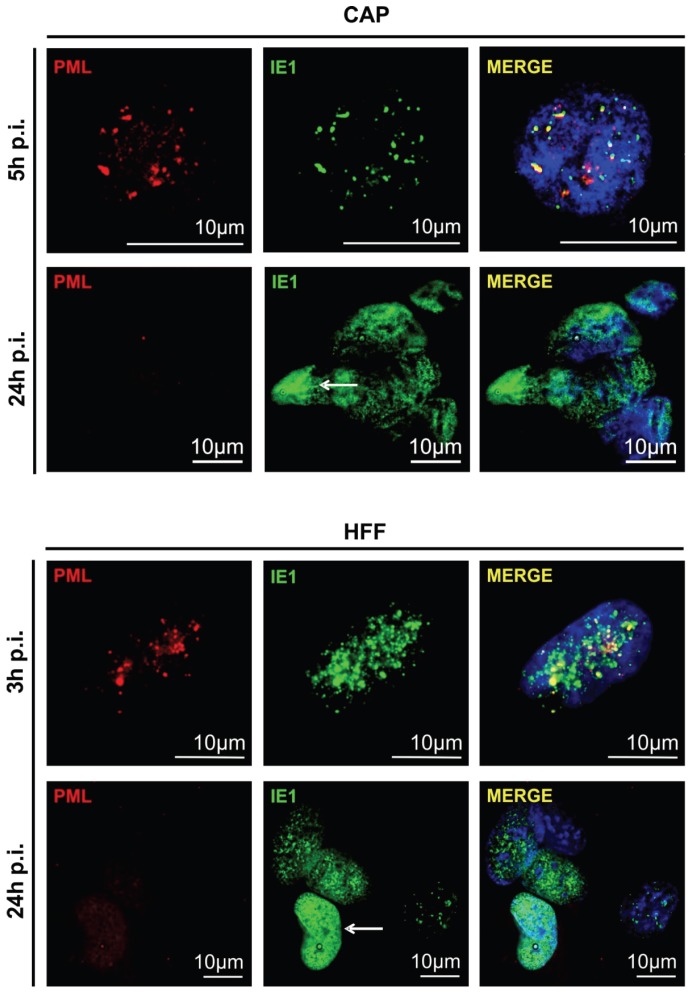
IF-analysis of the IE1-colocalization with and the subsequent dispersal of PML-bodies in CAP_adh__._ and HFF (control). Cells were infected with TB40/E-BAC7 at an m.o.i. of 2. Infected HFF were taken along as control. After 5 and 24 h (CAP_adh__._, upper panel) or 3 and 24 h (HFF, lower panel) of infection, samples were collected and subjected to IF-analysis using the IE1-specific monoclonal antibody p63-27 as well as a rabbit polyclonal antibody directed against PML. p63-27 binding was detected with an Alexa Fluor 488 labeled secondary antibody (green). PML was detected with an Alexa Fluor 546 labelled secondary antibody (red). For staining of the nuclei, DAPI (blue) was added to the samples. White arrows point to IE1-stained nuclei after dispersal of PML bodies.

Permissive HCMV infection of human fibroblasts is characterized by concomitant attempts of the host cell to silence incoming viral genomes through the attachment of a repressive chromatin structure, thereby shutting down viral gene expression. This is mediated by cellular proteins accumulating in specific nuclear structures, termed ND10-domains or PML bodies [50,51,52]. To avoid silencing of its genes, HCMV has developed strategies to counteract the attachment of repressive histones to viral promotors. This is considered pivotal to allowing viral replication. One such mechanism depends on the dissolution of ND10-domains by the de-SUMOylation of the scaffold protein PML by viral IE1 [53,54]. To test if PML bodies were dispersed in CAP_adh._, as they were in fibroblasts, double immunofluorescence staining was performed on both cell types, using IE1- and PML-specific antibodies. CAP_adh._ and HFF were infected with TB40/E-BAC7 at an m.o.i. of 2. Here, a TB40/E strain was used which does not express GFP. In both cases, infection initially led to partial detachment or rounding of infected cells, which is resolved by re-attachment at 3 h p.i. (HFF) or 5 h p.i. (CAP), respectively. Consequently, cells were stained at the earliest time point after re-attachment. Following fixation, cells were stained with the IE1-specific antibody [42] (using an Alexa Fluor 488 labelled secondary antibody; green; Figure 3) and with a PML-specific antiserum (using an Alexa Fluor 546 labelled secondary antibody; red; Figure 3). IE1 initially colocalized with PML in CAP_adh._, comparable with IE1 localization in HFF. At 24 h p.i., IE1 was evenly distributed throughout the nucleus, accompanied by a complete dispersal of PML in both CAP_adh._ and HFF (white arrows). These results showed that IE1-PML (ND10) interaction in CAP cells was comparable to that seen in permissive HFF.

### 3.3. Detection of Nuclear Replication Compartments and Late Protein pp28 in HCMV Infected CAP Cells

**Figure 4:**
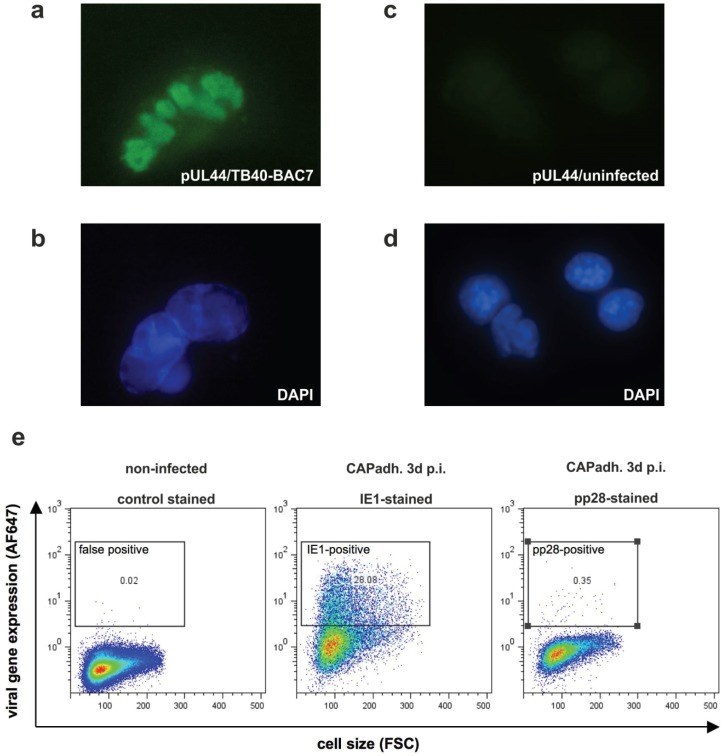
Analysis of nuclear replication compartment formation and of late gene expression in CAP cells. (**a**) analysis of nuclear replication compartment formation by indirect immunofluorescence staining of pUL44 localization in CAP_adh._, infected with TB40/E-BAC7 at an m.o.i. of 0.5 for five days; (**b**) DAPI staining of the nuclei of the cells from panel a; (**c**) staining of uninfected cells with a pUL44 specific antibody; (**d**) DAPI staining of the nuclei of the cells from panel c; (**e**) FACS analysis of IE1 and pp28 expression in CAP_adh._. Cells were infected with TB40/E-BAC4deltaUL5-9luc at an m.o.i. of 2. At 1.5 h after infection, the inoculum was removed. Cells were then incubated with 0.14 M NaCI, exposed to 0.1 M glycine (pH 3.0) for 1 min to inactivate residual virus and were subsequently washed with maintenance medium. Three day after infection, cells were collected, fixed with ethanol, and incubated with specific monoclonal antibodies. Cells were then incubated with an AF647 labeled anti-mouse specific secondary antibody. Unspecific binding of primary antibodies was excluded by incubation of infected cells with a mouse isotype control. Viral protein expression was measured by FACS. Results were depicted in viral gene expression against total cell size.

To investigate if viral nuclear replication compartments were formed in infected CAP cells, they were stained by indirect immunofluorescence using an antibody directed against the viral DNA-polymerase processivity protein pUL44. Antibodies against this protein are known to label the replication compartments. The structure became indeed visible in the nucleus of CAP_adh._, infected with TB40/E-BAC7 at an m.o.i. of 0.5 for five days. (Figure 4). This indicated that nuclear compartments that are considered to be essential for the initiation of viral DNA replication were formed in CAP cells. To address the question of whether late proteins were detectable in infected CAP cells, FACS analysis was performed on TB40/E-BAC4deltaUL5-9luc (a GFP neg. strain) infected CAP cells [43,55]. As an example, the late protein pp28 was examined. The protein was found in a very minor fraction of the cells, indicating that late viral gene expression was only moderately effective in HCMV infected CAP cells (Figure 4).

### 3.4. HCMV-DNA Is Replicated in CAP Cells

**Figure 5:**
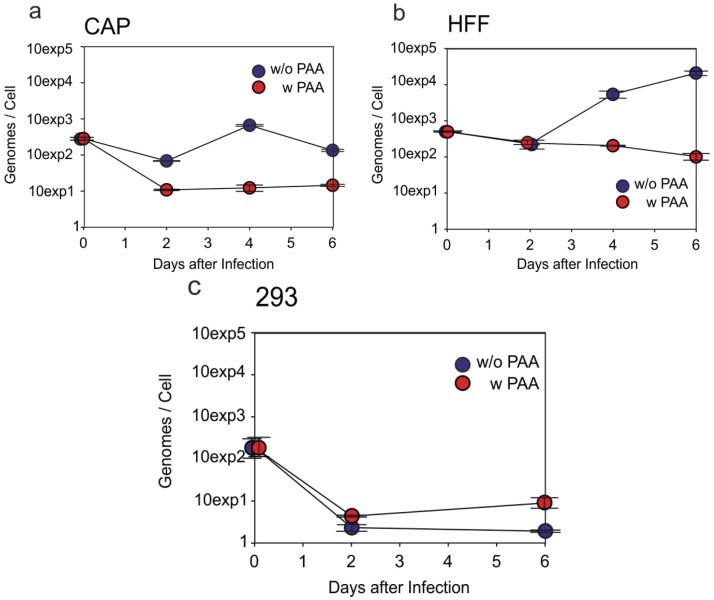
Quantitative PCR analysis of viral genome replication in CAP cells (**a**); HFF (**b**) and HEK293 (**c**) were taken along for control. Cells were infected with TowneUL130rep at an m.o.i. of 2.5. At l.5 h after the addition of virus, the inoculum was removed. The cells were washed with 0.14 M NaCl, exposed to 0.1 M glycine (pH 3.0) for 1 min to inactivate residual virus, and were washed with maintenance medium. Afterwards cells were either incubated with or without the addition of 0.25 mg/mL PAA. Cells were collected at the indicated time points after infection and tested using the ABI 7500 Fast Real-Time PCR Detection System. Results were calculated in genome copies per cell. Error bars represent means of three technical replicates.

FACS analyses had revealed low levels of late pp28 protein expression. To address the question of whether viral DNA replication, a prerequisite of late protein expression, was initiated in HCMV infected CAP cells, viral genome levels were determined by quantitative real time PCR. For this, CAP_adh._ were infected with TowneUL130rep at an m.o.i. of 2.5 (Figure 5a). HFF (Figure 5b) and HEK293 (Figure 5c) served as controls. Parallel incubation of cells with the viral DNA-polymerase inhibitor phosphonoacetic acid (PAA) served as negative control. Cells were collected at different time points after infection. Viral DNA was extracted from the cells and quantified using the ABI 7500 Fast real-time PCR detection system. Results were calculated in genome copies per cell. The HCMV genome levels dropped slightly at two days p.i. in infected CAP_adh._, but increased to roughly 1000 copies per cell at four days p.i. (Figure 5a). Compared to the negative control (PAA), genome levels were roughly 100-fold higher at four days p.i. Genome copy numbers dropped again at six days p.i. The DNA levels that were reached in CAP_adh._ were lower compared to those found in infected HFF (Figure 5b). No viral DNA replication was noted in infected HEK293 (Figure 5c). These cells do support virus entry and IE1 gene expression, but are known to be non-permissive for viral DNA replication [27]. Interestingly, these cells are transformed by the same adenoviral E1 gene functions as CAP cells [25,31], indicating that the expression of E1A and E1B in CAP cells does not generally block HCMV DNA replication. Taken together, these results provided proof that HCMV DNA replication was initiated in CAP cells.

### 3.5. HCMV Capsid and Virion Morphogenesis Is Supported by CAP Cells

**Figure 6:**
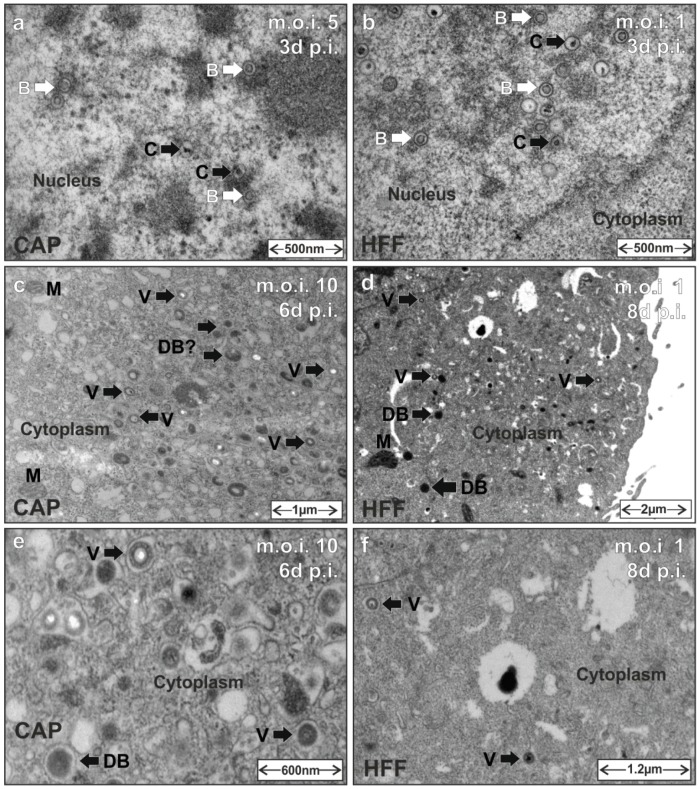
Transmission electron microscopy (TEM) of particle morphogenesis in HCMV infected CAP cells. HFF were taken along for control. Cells were infected with either TowneUL130rep at an m.o.i. of 5 (**a**) or 1 (**b**) or with TB40/E-BAC4deltaUL5-9luc at an m.o.i. of 10 (**c**,**e**) or 1 (**d**,**f**), respectively. Then, 1.5 h after the addition of virus, the inoculum was removed. The cells were washed with 0.14 M NaC1, exposed to 0.1 M glycine (pH 3.0) for 1 min to inactivate residual virus, and were washed with maintenance medium. At the indicated times after infection, cells were processed for TEM. B, B-capsids. C, C-capsids. V, virions. DBs, dense bodies. M, mitochondria.

A hallmark of HCMV infection is the formation of nuclear capsids during the assembly process. After nuclear capsid egress, viral morphogenesis continues in the viral assembly compartment in the cytoplasm of infected cells. To investigate if HCMV capsid and virion morphogenesis were supported by CAP cells, CAP_adh._ or HFF (control) were infected with either TowneUL130rep or a TB40/E derived strain. At three days p.i., six days p.i. or eight days p.i., cells where analyzed by transmission electron microscopy (TEM). Particles with a translucent core (B-capsids) and also with a condensed core (mature C-capsids, [56]) were found in three-day infected CAP_adh._ (Figure 6a). These structures corresponded to the particles found in the nuclei of HFF three days p.i. (Figure 6b).

At later stages of infection, viral particles were also found in the cytoplasm of infected CAP_adh._ (six days p.i.; Figure 6c,e). These particles resembled the virions that were detectable in the cytoplasm of HFF at eight days p.i. (Figure 6d,f). As the TB40/E (TB40/E-BAC4deltaUL5-9luc) strain used for this analysis does not produce high levels of DBs, the bulk production of such particles usually seen in laboratory strain infected fibroblasts was not detectable. Yet, in both CAP_adh._ and HFF, smaller electron dense structures became detectable that may be related to DBs. These data indicate that HCMV particle morphogenesis is fully supported by CAP_adh._. It has to be mentioned, however, that viral particles were found in roughly one of 100 CAP cells, inspected at low magnifications in TEM.

### 3.6. Viral Progeny Is Released from Infected CAP Cells

**Figure 7:**
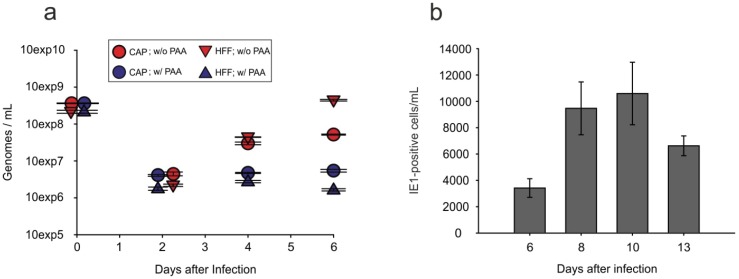
Viral genome release and release of infectious virus into the supernatant of infected CAP cells. (**a**) Quantitative PCR analysis of viral genome release from infected CAP_adh._, measured by quantitative PCR. CAP_adh._ and control HFF were infected with TowneUL130rep at an m.o.i. of 2.5. At l.5 h after the addition of virus, cells were washed with 0.14 M NaCI, exposed to 0.l M glycine (pH 3.0) for 1 min to inactivate residual virus, and were subsequently washed with maintenance medium. Afterwards, cells were either incubated with or without the addition of 0.25 mg/mL PAA. Cells were collected at two days, four days and six days after infection. Viral DNA was extracted and analyzed by real-time PCR. Results were calculated in genome copies per cell. Error bars represent means of three technical replicates; (**b**) Analysis of the release of infectious virus by CAP_adh._ through IE1-staining. Cells were infected with TB40/E-UL16EGFP at an m.o.i. of 10. Supernatants were collected and tested in serial dilutions in eight biological replicates on HFF for the number of IE1-positive cells. Error bars represent means of eight technical replicates.

TEM analysis suggested that HCMV progeny were produced in CAP_adh._. To test if progeny were released from the cells, viral genome copies were measured in the culture supernatants by quantitative real-time PCR. For this, CAP_adh._ and HFF were infected at an m.o.i. of 2.5 with TowneUL130rep and were treated with 0.14 M NaCl/0.1 M glycine (pH 3.0) for 1 min to inactivate residual virus after an infection period of 1.5 h. Cells were then incubated either in the presence or absence of PAA. Culture supernatant was collected at two days, four days and six days after infection. Viral DNA was extracted and analyzed by real-time PCR. Results were calculated in genome copies per mL. The results showed that viral genomes were released by infected CAP_adh._ (Figure 7a). This release was sensitive to PAA, excluding the possibility that input DNA was measured. Of note, the number of genomes found in the supernatant of CAP_adh._ was considerably lower than the copy number found in HFF supernatants.

To measure the release of infectious virions, supernatants of infected CAP cells were collected at different time points after infection. Titers of infectious virus were determined by measuring IE1-positive cells on indicator fibroblasts [42]. Infectious virus was indeed released from infected CAP_adh._ (Figure 7b). The titers were, however, roughly three orders of magnitude lower than those usually seen with culture supernatants from infected HFF [49]. Still, these results proved that CAP cells were fully permissive for HCMV replication and virus production.

### 3.7. Purification of Viral Particles from Infected CAP Cells

**Figure 8:**
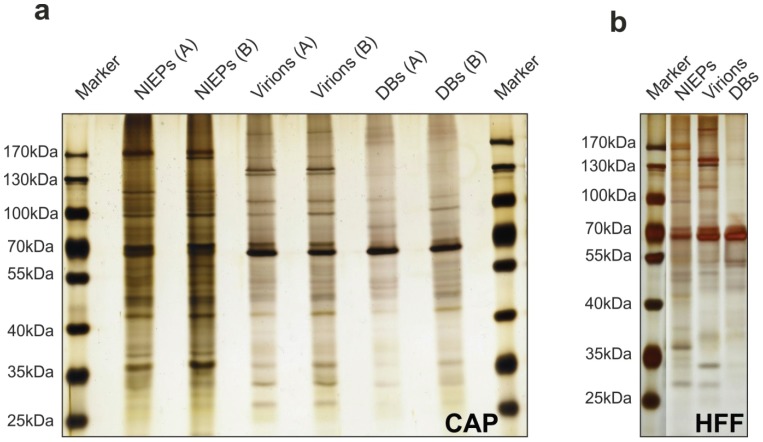
Gel analysis of purified viral particles released into the culture supernatant of adherent CAP cells and HFF (control). CAP_adh._ and HFF were infected with TowneUL130rep at an m.o.i. of 5 (CAP; A), 2.5 (CAP; B) or l (HFF). Culture supernatants were collected at seven days p.i. and were subjected to gradient centrifugation. Two micrograms of each of the fractions from the gradients were subsequently subjected to PAGE and silver staining. (**a**) NIEPs, Virions, and DBs from the two different purifications; (**b**) NIEPs, Virions, and DBs from the purification of particles from HFF supernatants.

DBs are an attractive basis for HCMV vaccine development. A major goal of this study was to investigate if CAP cells would be an appropriate cell substrate for DB-production. To test this, viral particles were purified from the culture supernatants of infected CAP_adh._, using standard ultracentrifugation protocols [57]. Typical bands, corresponding to DBs, NIEPs, and virions were detectable in the gradients of two independent purification experiments [49]. Two micrograms of each fraction were subjected to PAGE and silver staining (Figure 8). Patterns of viral proteins, corresponding to the pattern seen in the control (particles from HFF, Figure 8b), were found. These results suggest that CAP_adh._ release the three major HCMV particle types, including DBs that are also found in the supernatants of infected HFF cultures [44,57].

## 4. Discussion

There is a well-known discrepancy between the very efficient replication of HCMV in various cell types *in vivo* and the limited productivity of cell culture infection [22,23,58]. Only primary human fibroblasts serve as substrate for high-level progeny production of HCMV, whereas other primary or tumor derived human cell cultures show only limited HCMV replication. Cell lines immortalized by transformation with SV40 large T antigen or adenovirus E1A/E1B functions are notoriously resistant to HCMV replication [27,28]. Here, we present, for the first time, evidence that adenovirus E1A/E1B expression does not disable HCMV replication and that amniotic fluid cells, transformed with E1A and E1B, support the full replication cycle of HCMV.

Amniotic fluid (AF) contains multiple cell types derived from the developing fetus. A minor cell population shows characteristics of pluripotent stem cells and has attracted considerable attention for its potential use in stem cell therapy [59,60,61]. It appears unlikely that CAP cells are derivatives of that fraction, since culture conditions initially used for the generation of this cell line appear to be inappropriate for the growth of stem cells [31,59,61]. CAP cells may rather have originated from one of the two major classes of cell types that can be readily cultured from AF. These cell fractions show characteristics of either fibroblasts or of cells originating from fetal membranes [62]. As adherent CAP cells display an epithelial cell-like appearance rather than resembling fibroblasts, they most likely have originated from fetal membranes.

Several cell types of both maternal and fetal origin have been reported to be HCMV infected by looking at tissue specimens, explants and cell cultures [8,9,63,64,65]. Interestingly, amniotic epithelial cells have been shown to be susceptible to HCMV infection in culture [66]. In addition, Pereira *et al.* detected HCMV antigens in cytoplasmic vesicles of cells of the amniotic membrane in placental specimens from cases of primary and recurrent HCMV infection during pregnancy [8]. These membranes, facing the amniotic fluid, consist of polarized epithelial cells. Virus uptake was likely occurring from the amniotic fluid via their apical surface. Together, this suggests that CAP cells originated from a cell type with epithelial characteristics that was detached from the amniotic membrane or from other fetal membranes and that CAP cells have retained their susceptibility to HCMV despite transformation by E1A/E1B.

Initial HCMV infection and IE gene expression progressed effectively in CAP cells. This indicates that CAP cells express surface receptors for HCMV and that entry proceeds swiftly. It is unclear if infection of CAP cells occurred via membrane fusion at the cell surface or by endocytosis (for a review, see [67]). Upon EM-analysis, particles were visible in structures that resembled those seen in the process of macropinocytosis [49]. However, it remains unclear if these structures were responsible for HCMV penetration into CAP cells.

Interestingly, CAP cells could also be infected by HCMV laboratory strains. These strains do not express the pentameric complex consisting of gH/gL/gpUL128-131A. Opposed to EC or epithelial cells, the pentamer is thus not essential for CAP infection. It has been recently reported that membrane fusion, mediated by the viral gB, may proceed in the absence of the pentamer. Thus, the pentamer may not be involved in the regulation of fusion, as previously suggested, but may serve as an alternative function during adsorption and penetration [68]. This function is not yet known. It may be rate limiting when comparing infection of CAP cells by pentamer-positive *vs.* pentamer-negative strains. CAP cells may thus be an interesting cell substrate to approach this unknown, pentamer-dependent process during HCMV infection.

It remains unclear at this point, why CAP cells are successful in initiating HCMV DNA replication and progeny production, while other E1A/E1B transformed cells like HEK293 are not (see Figure 6). Small tumor viruses such as adenoviruses or SV40 express early proteins that interact with the retinoblastoma tumor suppressor protein (Rb). This leads to release of the transcription factors E2F and DP from a complex with Rb (reviewed by [69]). E2F and DP are key regulators that drive the cell into the S-phase, a mechanism beneficial for SV40 or adenovirus replication. In contrast, HCMV infection induces an initial halt of the cell cycle in G0/G1 in fibroblasts, which is considered a prerequisite for the initiation of viral DNA replication (reviewed by [70]). It remains unclear at this point if HCMV is able to halt the cell cycle in G0/G1 in CAP cells as efficiently as it does it in HFF.

Although viral genome replication was initiated in CAP cells, DNA synthesis appeared to be less efficient compared to what was seen in fibroblasts. IE genes, which are essential for efficient DNA replication, were expressed at high levels, rendering limitations at this stage unlikely. The expression of pUL44 and the formation of nuclear replication compartments were detectable in CAP_adh._, indicating that the processes needed for the initiation of viral DNA replication commenced. We cannot exclude, however, that one or more of the early viral proteins that are required for the assembly of the viral polymerase complex [71] were insufficiently synthesized and thus rate limiting for viral DNA replication. A more detailed analysis of the expression levels of all the viral proteins required for viral DNA replication, e.g., by mass spectrometry, is necessary to approach this issue.

Particle morphogenesis of HCMV was supported by CAP cells. However, viral particles were found in only a limited number of cells, inspected by TEM. This indicated that either a sub-fraction of CAP cells supported particle formation or, alternatively, the small fraction of initially uninfected cells overgrew infected cells at late stages after infection, thus representing the majority of cells at that time. In any case, TEM analysis proved the formation of B- and mature, DNA containing C-Capsids in the nucleus of some CAP cells. In addition, mature virions and DB-like structures were seen in the cytoplasm of infected CAP cells. These particles were found to be embedded in a region together with numerous membranous structures, likely reflecting the cytoplasmic viral assembly compartment with modified vesicular structures from the secretory apparatus [72,73]. DBs could be purified from TowneUL130rep infected CAP_adh._, demonstrating that DB-morphogenesis did occur. Still, the amount of DBs was low. Production of these particles on CAP cells for vaccine purposes is possible but clearly needs targeted optimization of culture conditions. Taken together, these results showed that the complicated process of particle assembly in infected cells progressed in a coordinated fashion in CAP cells to finally result in virion and DB formation.

CAP cells released progeny virions that were suitable to infect HFF. However, the infectivity was limited and roughly three orders of magnitude lower than that normally seen after infection of fibroblasts. Surprisingly, the rates of viral DNA release into the culture supernatant differed by only one order of magnitude, indicating a high particle to infectivity ratio for CAP-virus, compared to HFF-virus. This suggests that particle morphogenesis was only moderately efficient in CAP cells. In line with this, only very little of the late viral protein pp28 was found in CAP cells. In addition, secondary viral spread in CAP cultures did not seem to occur; infection of CAP cells with a GFP expressing variant of HCMV did not lead to focus formation and the GFP signal was lost in the course of cell passaging. This is somewhat resembling the situation in EC, where virions released into the supernatant are infectious for fibroblasts, but hardly re-infect EC [41]. However, focal spread is seen in EC cultures, which was not detectable in CAP cells. Further analysis of the composition of virions derived from CAP-derived HCMV is required to determine the difference between input and progeny in order to identify viral proteins that limit CAP cell spread.

CAP cells have been designed for the production of recombinant proteins, of adenovirus-based vectors for gene therapy, and for viral vaccines [26,31]. They are susceptible to infections with influenza virus, with RSV, and with poliovirus [32,33,34]. Here, we show that HCMV infected CAP cells do also release DBs. Because of their exceptional immunogenicity, DBs are a prime candidate for HCMV vaccine development [14,18,19]. Therefore, CAP cells are an attractive cell substrate for the production of DBs regarding vaccine development.

## 5. Conclusions

Based on our data, we conclude: (1) The expression of adenovirus E1A/E1B in stable cell lines is not generally repressive to HCMV replication. (2) Amniotic epithelial cells, transformed with E1A/E1B (CAP-cells) support the full replication cycle of HCMV. (3) Both virions and dense bodies of HCMV are released from infected CAP-cells. (4) CAP-cells are a promising cell substrate for vaccine production based on dense bodies.
